# BaTiO_3_-Based Electrocaloric Materials—Recent Progresses and Perspective

**DOI:** 10.3390/ma18174190

**Published:** 2025-09-06

**Authors:** Yi Tang, Xiang Niu, Yuleng Jiang, Junxi Cao, Junying Lai, Houzhu He, Jianpeng Chen, Xiaodong Jian, Sheng-Guo Lu

**Affiliations:** 1 School of Materials and Energy, Guangdong University of Technology, Guangzhou 510006, China; 3122005244@mail2.gdut.edu.cn (Y.T.); 2112325141@mail2.gdut.edu.cn (Y.J.); 3122005505@mail2.gdut.edu.cn (J.C.); 2112202115@mail2.gdut.edu.cn (J.L.); 3117005851@mail2.gdut.edu.cn (H.H.); 3122005338@mail2.gdut.edu.cn (J.C.); jianxiaodong@gdut.edu.cn (X.J.); 2 School of Integrated Circuits, Guangdong University of Technology, Guangzhou 510006, China; 3 Science and Technology on Reliability Physics and Application of Electronic Component Laboratory, The 5th Electronics Research Institute of the Ministry of Industry and Information Technology, Guangzhou 510610, China

**Keywords:** BaTiO_3_-based, ceramics, lead-free, electrocaloric effect, strategies

## Abstract

BaTiO_3_ (BT)-based lead-free ceramics are regarded as highly promising candidates for solid-state electrocaloric (EC) cooling devices due to their large spontaneous polarizations, shiftable Curie temperatures, and environmental friendliness. This review summarizes recent progresses in the design and optimization of BT-based EC ceramics. Key aspects include thermodynamic principles of the EC effect (ECE); structural phase transitions; and strategies such as constructing relaxor ferroelectrics, multi-phase coexistence, etc. Finally, future research directions are proposed, including the exploration of local microstructural evolution, polarization flip mechanisms, and bridging material design and device integration. This work aims to provide insights into the development of high-performance BT-based materials for solid-state cooling devices.

## 1. Introduction

With the rapid advancement of electronic components, particularly emerging technologies such as high-power integrated circuit chips, high-precision sensors and actuators, quantum computing devices, and new fuel automobiles, the demand for miniaturized thermal management in integrated, compact systems has grown remarkably. Traditional refrigeration technologies face challenges such as high energy consumption and poor integrability, driving researchers to seek more efficient and environmentally friendly solutions for next-generation small-scale refrigerations.

Currently, major novel refrigeration technologies include thermoelectric (TE) (semiconductor-based) [1], magnetocaloric (MC) [2], electrocaloric (EC) [3], elastocaloric (eC) [4], and barocaloric (BC) [5] refrigerations. Among them, TE refrigeration—a mature technology based on the Peltier effect—utilizes the TE properties of thermoelectric materials for temperature regulation. Its advantages involve no moving parts, quiet operation, high reliability, and suitability for precise temperature control in small electronic devices. However, its cooling efficiency is limited by unavoidable Joule heating during operation, resulting in higher energy consumption and hindering large-scale applications. MC, EC, eC, and BC refrigerations are collectively termed “caloric refrigeration technologies”, which achieve cooling through external stimuli such as magnetic field, electric field, uniaxial stress, or hydrostatic pressure. MC refrigeration drives temperature changes via a magnetic field variation but requires a strong magnetic field for effective cooling, posing challenges for miniaturization, integration, and commercialization due to the high cost of permanent magnets and limited DC magnetic field [6]. BC and eC technologies suffer from poor fatigue resistance in materials, limiting their widespread use [6,7,8].

In contrast, EC refrigeration is regarded as a highly promising solid-state cooling technology, particularly for on-chip thermal management [9]. Owing to its high cooling efficiency, low cost, excellent fatigue resistance, environmental friendliness, and compatibility with device miniaturization and system integration, EC refrigeration has attracted widespread attention from researchers worldwide. The core principle of EC refrigeration lies in the EC effect (ECE) [10]. The ECE is an intrinsic phenomenon of ferroelectrics described by the thermodynamic theory. Under an applied electric field, the polarization state of ferroelectrics undergoes changes accompanied by an isothermal entropy change (∆S) or an adiabatic temperature change (∆T) in the material. When an electric field is applied, the electric dipoles within the material transition from a disordered state to an ordered state, leading to a reduction in the polarization entropy. In an adiabatic environment, where the total entropy of the material remains constant, the lattice vibrational entropy must correspondingly increase, resulting in a temperature rise. Conversely, upon removal of the external electric field, the dipoles revert to a disordered state, the polarization entropy increases, and the lattice vibrational entropy reduces; consequently, the material’s temperature declines.

The ECE was first theorized in 1878 by William Thomson (Lord Kevin), who predicted its existence and its inverse relationship with the pyroelectric effect [11,12]. Almost half a century later, Kobeko et al. [13] experimentally observed the ECE in Rochelle salt (NaKC_4_H_4_O_6_·4H_2_O) in 1930, but they did not show the data. Subsequently, in 1963, Wiseman and Kuebler measured a ∆T of 0.0036 K in Rochelle salt [14]. Over the following decades, the ∆T achieved in EC materials remained insufficient for practical applications. A breakthrough occurred in 2006 when Mischenko et al. [15] reported a 12 K ∆T in PbZr_0.95_Ti_0.05_O_3_ thin films using an indirect method. In 2008, Neese et al. [16] also demonstrated a giant ECE of 12 K in ferroelectric polymer (P(VDF-TrFE-CFE)) films via an indirect calculation. These findings ignited global research interest in ECE. However, both results were derived using the indirect method. In 2010, Lu et al. [17] directly measured the giant ECE of ~12 K (@ 169.8 MV/m, 318 K) in P(VDF-TrFE-CFE) 59.2/33.6/7.2 mol% terpolymer films and 5 wt% P(VDF-CTFE) 91/9 mol% copolymer films using a thermistor-based direct measurement, revealing significant discrepancies between the direct measurements (~3.6 K @ 70 MV/m, room temperature) and the Maxwell relation-derived indirect calculation (~0.87 K @ 70 MV/m, room temperature). This underscores the necessity of a direct measurement method for obtaining accurate ECE data.

Currently, mature ferroelectric material systems include barium titanate (BT)-based [18], bismuth sodium titanate (Na_0.5_Bi_0.5_TiO_3_, NBT)-based [19], bismuth ferrite (BiFeO_3_, BF)-based [20], sodium niobate (NaNbO_3_, NN)-based [21], potassium sodium niobate (K_0.5_Na_0.5_NbO_3_, KNN)-based [22], and lead (Pb)-based materials [15]. Recent advances have identified promising EC materials systems in ferroelectric/antiferroelectric ceramics, single crystals, thin/thick films, and polymer films, with notable breakthroughs in device applications. For practical solid-state electrocaloric refrigeration, an ideal material should contain (a) superior ECE across a broad temperature range near room temperature; (b) excellent thermal stability, frequency stability, and fatigue resistance; and (c) structural simplicity and ease of miniaturization/integration.

The key to practical applications of EC refrigeration lies in seeking materials with a large ECE, suitable refrigeration units, and precisely designed EC devices. BT is one of the most widely studied EC materials due to its strong spontaneous polarization, pronounced ferroelectric–paraelectric phase transition slightly higher than room temperature, and its non-toxic, lead-free composition. Its relatively high dielectric constant and excellent chemical stability make it an ideal candidate for EC applications. The advantages and disadvantages of BT as an EC material are discussed in detail in Section 4.

**Figure 1:**
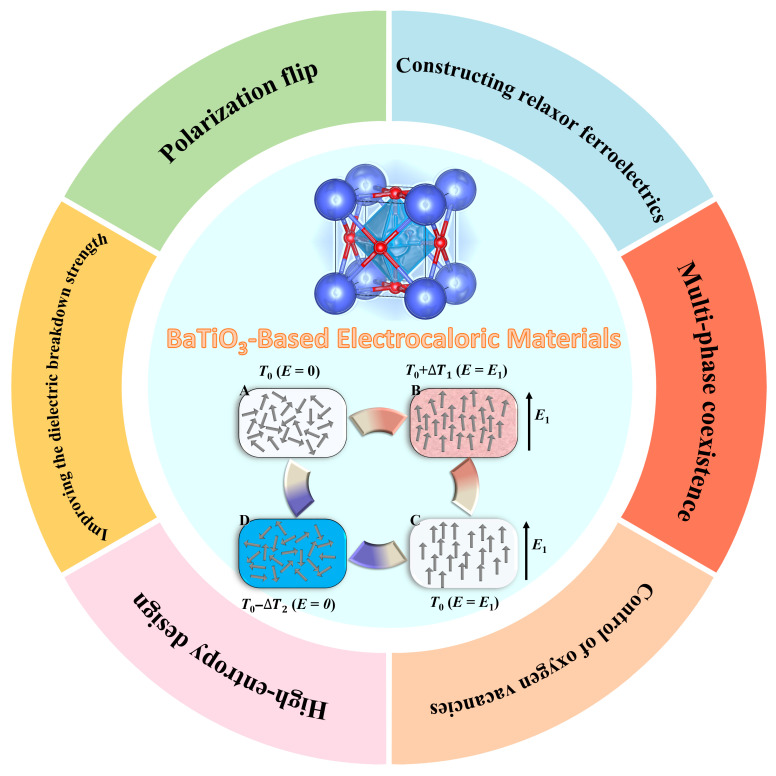
Overview of the progresses and key strategies in BaTiO_3_-based EC materials.

In this review, we systematically summarize the recent advances in BT-based EC ceramics, focusing on their intrinsic advantages, strategies, and performance optimization (Figure 1). We begin by introducing the fundamental physical mechanisms underlying the ECE, including the indirect calculation method and direct measurement methods. Subsequently, we discuss the rationale for selecting BT-based ceramics. Key strategies to enhance ECE are comprehensively reviewed. Finally, we present perspectives on future directions, highlighting remaining challenges and opportunities in the development of high-performance BT-based EC materials for next-generation solid-state cooling applications.

## 2. Indirect Calculation Method

### 2.1. Maxwell Relations

The Gibbs free energy of a dielectric material can be expressed as a thermodynamic characteristic function dependent on the temperature (*T*), entropy (*S*), stress (*X*), strain (*x*), electric field (*E*), and electric displacement (*D*). For electrocaloric materials, the Gibbs free energy can be written as

(1) G=U−TS−Xx−ED,

where $D=\varepsilon_{0}E+P=(\varepsilon_{0}+\varepsilon_{r})E$, with $\varepsilon_{0}$ denoting the vacuum permittivity ($\varepsilon_{0}$ = 8.854 × 10^−12^ F/m) and $\varepsilon_{r}$ indicating the relative permittivity. Since $\varepsilon_{r}$≫ $\varepsilon_{0}$, for ferroelectric materials, *D* ≈ *P*. Equation (1) can be simplified into

(2) G=U−TS−Xx−EP,

The total differential form of *G* is

(3) dG=−SdT−xdX−PdE,

At certain *T*, *X*, and *E*, the *S*, *x*, and *P* can be derived as

(4) $$S=-{\left(\frac{\partial G}{dT}\right)}_{X,E},$$

(5) $$x=-{\left(\frac{\partial G}{dX}\right)}_{T,E},$$

(6) $$P=-{\left(\frac{\partial G}{dE}\right)}_{X,T},$$

Since the Gibbs free energy *G* is continuous and differentiable under a constant *X*, the partial derivatives yield

(7) $${{\left(\frac{\partial S}{\partial E}\right)}_{T}=\left(\frac{\partial^{2}G}{\partial T\partial E}\right)}_{T}={\left(\frac{\partial^{2}G}{\partial E\partial T}\right)}_{E}={\left(\frac{\partial P}{\partial T}\right)}_{E},$$

(8) $${\left(\frac{\partial S}{\partial E}\right)}_{T}={\left(\frac{\partial P}{\partial T}\right)}_{E},$$

where ${\left(\frac{\partial P}{\partial T}\right)}_{E}$ represents the pyroelectric coefficient, indicating that the ECE is the inverse of the pyroelectric effect. The ∆S can be expressed as [16]

(9) $$∆S=\int_{E_{1}}^{E_{2}}{\left(\frac{\partial P}{\partial T}\right)}_{E}dE,$$

where *E*_1_ and *E*_2_ denote initial and final electric fields. And since $∆T=-\frac{T}{C_{p}\rho }\cdot ∆S$, the ∆T can be written as [16]

(10) $$∆T=-\frac{T}{C_{p}\rho }\int_{E_{1}}^{E_{2}}{\left(\frac{\partial P}{\partial T}\right)}_{E}dE,$$

where *T* is temperature (unit is K), *C_p_* is the specific heat capacity on *T*, and *ρ* is the density of the material.

While Maxwell relations have been widely applied, discrepancies still exist between indirect and the direct measurements. Assumptions such as constant *C_p_* (neglecting its dependence on the temperature and the electric field) might bring about errors. Additionally, the Maxwell relations should be modified for relaxor ferroelectrics and ferroelectrics with a first-order phase transition due to the distribution of polar nano-regions for the former and the discontinuity of polarization as a function of temperature for the latter [23].

### 2.2. Landau Phenomenological Theory

Let *P* indicate the order parameter; the Gibbs free energy using Landau theory can be expressed as

(11) $$G=G_{0}+\frac{1}{2}\alpha P^{2}+\frac{1}{4}AP^{4}+\frac{1}{6}\gamma P^{6}-EP,$$

Here, α, A, and γ are thermodynamic coefficients; *α* is related to the temperature, i.e., $\alpha =\beta (T-T_{c})$ (*β* is the phenomenological coefficient independent of temperature); A and *γ* are the temperature-independent coefficients. For the first-order phase transition, A < 0 and *γ* > 0; for the second-order transition, A > 0 and *γ* = 0. *G*_0_ is the Gibbs free energy when material is in the paraelectric phase, in which the Curie–Weiss law is followed; then,

(12) $$\beta =\frac{1}{\varepsilon_{0}C},$$

Here, *C* is the Curie constant. The Gibbs free energy becomes

(13) $$G=G_{0}+\frac{1}{2}\beta (T-T_{C})P^{2}+\frac{1}{4}AP^{4}+\frac{1}{6}\gamma P^{6}-EP,$$

Differentiating *G* with respect to *T* yields

(14) $$∆S=-{\left(\frac{\partial G}{\partial T}\right)}_{E}=-\frac{1}{2}\beta \left[P^{2}\left(E_{2},T\right)-P^{2}\left(E_{1},T\right)\right],$$

In practical applications, for maximum entropy change (*E*_1_ = 0, *P*^2^ (*E*_1_, *T*) = 0), then

(15) $$∆S=-\frac{1}{2}\beta P^{2}(E,T),$$

(16) $$∆T=\frac{1}{2}\frac{T}{C_{p}\rho }{\beta P}^{2}(E,T),$$

Moreover, differentiating Equation (13) with respect to *P* in a constant electric field*,* and neglecting the higher-order terms, the relationship between the electric field *E* and the polarization *P* can be obtained as follows:

(17) $$E=\beta \left(T-T_{c}\right)P+AP^{3},$$

Substituting Equation (16) into Equation (17), one can obtain [23,24]

(18) $$\frac{\beta \left(T-T_{c}\right)}{{\left(-\frac{\beta T}{2C_{p}\rho }\right)}^{\frac{1}{2}}}∆T^{\frac{1}{2}}+\frac{A}{{\left(-\frac{\beta T}{2C_{p}\rho }\right)}^{\frac{3}{2}}}∆T^{\frac{3}{2}}=E,$$

Equation (18) reveals that under lower electric fields, ∆T is proportional to *E*^2^; under higher electric fields, ∆T is proportional to $E^{\frac{2}{3}}$ [24,25]. As can be inferred from Equations (15) and (16), to achieve larger values of ∆S and ∆T, the EC material should possess a larger *β* and a larger *P*, which is usually associated with a higher breakdown electric field.

### 2.3. Electrocaloric Strength (∆T/∆E)

In practical electrocaloric device applications, driving voltages range from a few volts to tens of volts, up to standard AC voltages such as 220 V or 380 V, and even higher. Although multilayer ceramic capacitor structures can effectively reduce the voltage under the same electric field due to the smaller thickness of single ceramic layer, the long-term operational voltage is typically selected as 30–50% of the breakdown voltage (based on the field) to ensure the device reliability for operation for over a hundred thousand cycles. Enhancing the ∆T at a lower electric field would significantly improve the cyclic reliability and stability of devices (i.e., achieving superior ECE with smaller voltage sources). Specifically, a higher electrocaloric strength enables superior electrocaloric performance under identical electric fields. As the electric field increases, the polarization of materials gradually saturates, leading to a slower increase in polarization per unit field, which implies a corresponding deceleration in the growth of polarization entropy per unit field. Consequently, the electrocaloric strength of a material typically increases initially and then decreases with the rising electric field (provided no dielectric breakdown or electrical degradation occurs), consistent with the derived conclusion using Equation (18). Improving the electrocaloric strength remains a key strategy for identifying materials with high ECEs. Traditionally, the electrocaloric strength is calculated by deriving ∆T (obtained via indirect or direct methods) via ∆T/∆E. However, this approach fails to directly correlate with the dielectric or the ferroelectric properties. To address this issue, Lu et al. [26] extended the Landau phenomenological theory to derive a novel formula for calculating the electrocaloric strength. In the paraelectric phase, where the spontaneous polarization is zero and the adiabatic temperature change vanishes, differentiating Equation (16) with respect to the electric field yields the electrocaloric strength expression [26]:

(19) $$\frac{dT}{dE}=\frac{\beta \varepsilon_{0}\varepsilon_{r}TP}{C_{p}\rho },$$

It can be observed that the electrocaloric strength is a function of temperature, dielectric constant, specific heat capacity, and polarization in a high electric field. Notably, *β* is a physical quantity associated with the electric field. When calculating *β* using the DC biased dielectric constant as a function of temperature under different electric fields, the resulting quantitative results are more accurate. Additionally, *C_p_* is a physical quantity related to both temperature and the electric field. To accurately represent the material’s specific heat capacity under a bias field, it is necessary to measure its variation with temperature. Moreover, since the electric field has a significant tuning effect on the dielectric constant, using the dielectric constant under different fields in the above formula calculations is more accurate and reasonable.

In summary, based on the Maxwell relation, Landau phenomenological theory, and the electrocaloric strength calculation formula, to achieve an excellent ECE, EC materials should possess a large phenomenological coefficient, large enough polarization variation, a high pyroelectric coefficient, and a higher breakdown field strength.

## 3. Direct Measurement Methods

**Figure 2:**
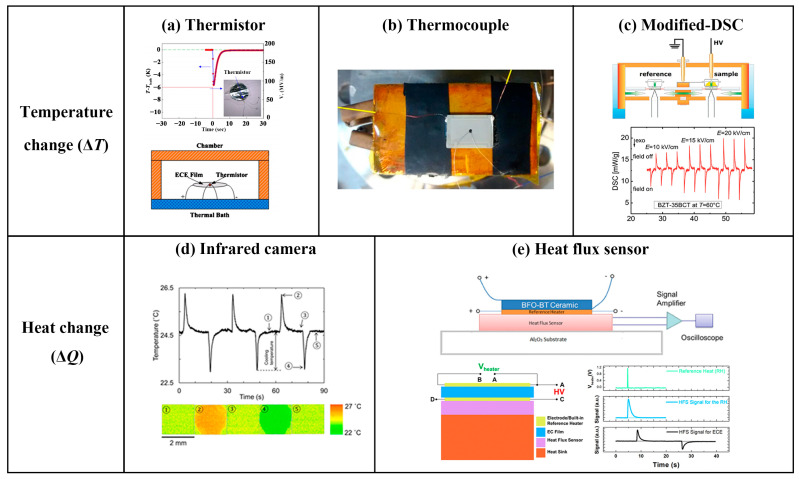
Direct measurements. (**a**) Thermistor method [17]; (**b**) thermocouple method [29]; (**c**) modified-DSC method [31]; (**d**) infrared thermography [32]; (**e**) heat flux sensor method [20,34].

For electrocaloric materials, direct measurement methods offer greater applicability, accuracy, and research value than traditional indirect calculation methods. As shown in Figure 2, common direct measurement methods include using a thermistor [17,27], thermocouple [28,29,30], modified differential scanning calorimetry (DSC) [31], infrared thermography [32,33], and heat flux sensor [20,34].

These methods can reveal the evolution of the ECE with the electric field and temperature, providing more reliable experimental foundations for developing high-performance ECE refrigeration materials. Unlike indirect approaches, direct measurement methods are applicable to all EC materials. A critical consideration for these methods is the presence of distinct heat dissipation pathways. Achieving an adiabatic environment and minimizing heat loss are pivotal for ensuring measurement precision and accuracy.

For thermocouple and thermistor-based direct measurements, temperature signals are first converted into voltage signals, which are then transformed into corresponding temperature readings via transmitters. Usually, the thermocouple has a thermal load when measuring the temperature due to the electronic transportation; the thermistor has a slower response time (e.g., for NTC thermistors, the thermal response time (∆*T*/*T*_env_ = 63.2%) is about 1~15 s). Modified-DSC and heat flux meter methods directly measure the heat released or absorbed by a material during the application or removal of an electric field at a specific temperature. The resulting exothermic/endothermic peaks are integrated to quantify the heat changes, which are subsequently converted into temperature variations based on the relationship between the entropy change ∆S and the ∆T in terms of *T*·∆S = −*C_P_*·∆T. Infrared thermography measures the surface temperature changes using an infrared thermal camera, where the measurement accuracy relies critically on the emissivity calibration and the construction of a blackbody environment.

## 4. Why Choose BT as an Electrocaloric Material?

BT-based ceramic is one of the most promising lead-free EC materials, owing to its favorable intrinsic properties and structural tunability. The recently reported ECEs of BT-based materials are summarized in Table 1 [35,36,37,38,39,40,41,42,43,44,45,46,47,48,49,50,51,52,53,54,55,56,57,58,59,60,61,62,63,64,65,66,67,68,69,70,71,72,73,74,75,76,77,78,79,80,81,82,83,84,85,86,87,88,89,90,91], highlighting their great potential for solid-state cooling applications. The following advantages make BT a particularly attractive candidate.

**Figure 3:**
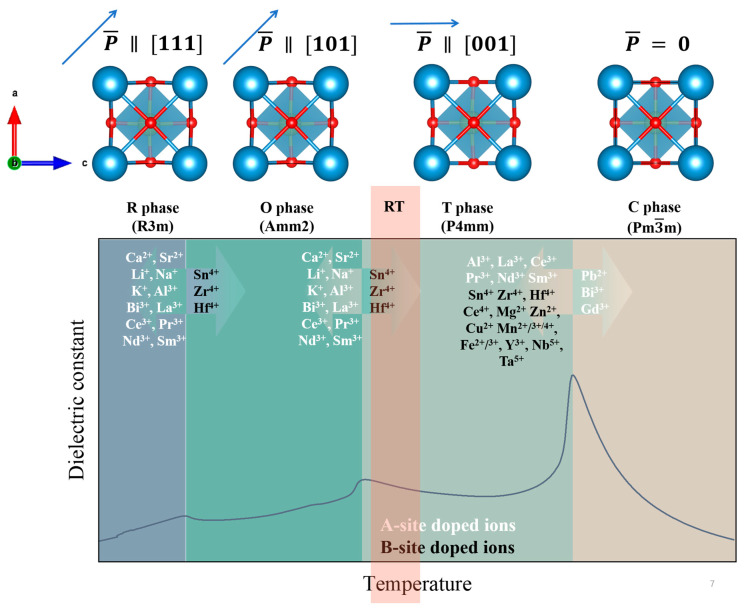
Temperature- and dopant-dependent phase structure evolution.

1. Large spontaneous polarization. Pristine BT exhibits a relatively large spontaneous polarization, which is crucial for achieving a large ECE. For instance, a pure BT single crystal exhibits a saturation polarization of ~26 μC/cm^2^ in the tetragonal phase at room temperature [92], providing a strong basis for polarization entropy change under an applied electric field.
2. Rich and well-defined phase transitions. The pristine BT unit cell (Figure 3) belongs to a perovskite ABO_3_ structure, where Ba^2+^ occupies the A sites, Ti^4+^ occupies the B sites, and O^2−^ occupies the six face centers. Due to the temperature-induced symmetry’s variation, pristine BT undergoes a series of well-characterized phase transitions with the increasing temperature, i.e., rhombohedral (R, R3m space group, spontaneous polarization direction along [1,1,1]), orthorhombic (O, Amm2 space group, spontaneous polarization direction along [1,0,1]), tetragonal (T, P4mm space group, spontaneous polarization direction along [0,0,1]), and finally cubic (C, Pm$\bar{3}$m space group, paraelectric). These transitions can be clearly observed in the dielectric constant versus temperature spectra, with peaks corresponding to R–O (~−90 °C, $T_{R-O}$), O–T (~0 °C, $T_{O-T}$), and T–C (~120 °C, Curie point, $T_{C}$) transitions, as shown in Figure 3. Generally, *T_C_* is especially important for the ECE due to the largest pyroelectric coefficient (d*P*/d*T*) near *T_C_*. Based on these temperature-induced phase transitions, one can obtain the enhanced dielectric and ferroelectric properties by adjusting the phase transitions and merging the various phase transitions. The fundamental mechanisms and effects of specific dopants are discussed below in detail.
3. Large phase transition enthalpy. Pristine BaTiO_3_ exhibits a phase transition enthalpy of approximately 900 J/kg, characterized by a first-order phase transition feature [93]. Under the influence of the electric field and the temperature, this enthalpy can be modulated. Although doping may slightly alter the phase transition enthalpy, when the doping concentration is low, the combined effect of phase transition enthalpy and the ECE can lead to an enhanced isothermal entropy change and adiabatic temperature change.
4. Stability, environmental friendliness, and low cost. As a commonly used composition in industrialized multilayer ceramic capacitors (MLCCs), BT exhibits excellent chemical stability without volatile constituents. In addition, as a lead-free material, it is environmentally friendly, and its raw materials are significantly less expensive compared to lead-based counterparts.

However, there are still many challenges in using pure BT for direct use in practical EC devices:

1. High Curie temperature. Ferroelectrics exhibit a large pyroelectric coefficient near their Curie temperatures. However, the $T_{C}$ of pristine BT is ~120 °C, which is far above room temperature. As a result, a pronounced ECE can only be achieved in the vicinity of 120 °C.
2. Sharp first-order phase transition. Pristine BT exhibits a typical first-order ferroelectric–paraelectric phase transition, leading to a large but narrow EC response localized near 120 °C. This is unfavorable for applications requiring a broad operating temperature range.

Although pristine BT exhibits several issues and challenges, its phase transition temperature, phase composition, and relaxor ferroelectric behaviors can be readily tailored using compositional modifications. A dopant-induced “peak-shifting effect” is usually accompanied by oxygen octahedral distortions, which in turn alter the phase structure, grain size, formation of secondary phase, and other microstructural features (e.g., superlattice)—all of which may significantly influence the ECE.

In general, A-site doping, B-site doping, or co-doping at both A- and B-sites are commonly employed strategies. A fundamental principle of doping is to select doping ions with ionic radius and electronegativity similar to the host ions. In the perovskite structure, the A-site cation typically exhibits a coordination number (CN) of 12, while the B-site cation has a CN of 6. Commonly used doping ions for each site are summarized in Table 2 and Table 3. The following section briefly introduces the effects of typical A-site and B-site ions on the phase transition behavior and relaxor characteristics of the material.

1. A-site doping. Generally, monovalent (Li^+^ [94], Na^+^ [95], K^+^ [96], etc.), divalent (Ca^2+^ [97], Pb^2+^ [98], Sr^2+^ [84], etc.), and trivalent (Bi^3+^ [99], La^3+^ [100], Ce^3+^ [101], Sm^3+^ [57,72], etc.) ions are often introduced at the A-sites. Doping ions introduces compositional and structural fluctuations, which lead to reductions in the $T_{C}$, $T_{O-T}$, and $T_{R-O}$ transition temperatures while also enhancing their relaxor ferroelectric behaviors, as shown in Figure 3. Notably, only Pb^2+^, Gd^3+^, and Bi^3+^ are capable of shifting $T_{C}$ toward higher temperatures [102,103,104,105]. In addition, Ca^2+^ can effectively stabilize the ferroelectric phase, resulting in negligible changes to $T_{C}$. However, both $T_{O-T}$ and $T_{R-O}$ decrease progressively with increasing Ca^2+^ content.
2. B-site doping. Ions such as Zr^4+^, Sn^4+^, and Hf^4+^ [18,37,82,106]—with similar radii and valences to Ti^4+^ (0.605 Å)—are commonly substituted at the B sites. These dopants typically reduce $T_{C}$ but enhance the lower phase transition temperatures ($T_{R-O}$, $T_{O-T}$) without significantly breaking the long-range ferroelectric ordering. Other dopants, such as Ce^4+^, Mg^2+^, Mn^2+/3+/4+^, Y^3+^, Nb^5+^, and Ta^5+^ [101,107,108], have also been used, though many tend to degrade the ferroelectricities and broaden the phase transitions, resulting in diffuse EC responses.

These chemical modifications enable precise control over BT’s phase transition behaviors and functional properties, allowing for the design of materials with enhanced and tunable ECE across a wider temperature range near room temperature.

## 5. Strategies

To achieve a high EC performance across a broad temperature range, the following design strategies have been adopted by researchers worldwide.

### 5.1. Constructing Relaxor Ferroelectrics (RFEs)

**Figure 4:**
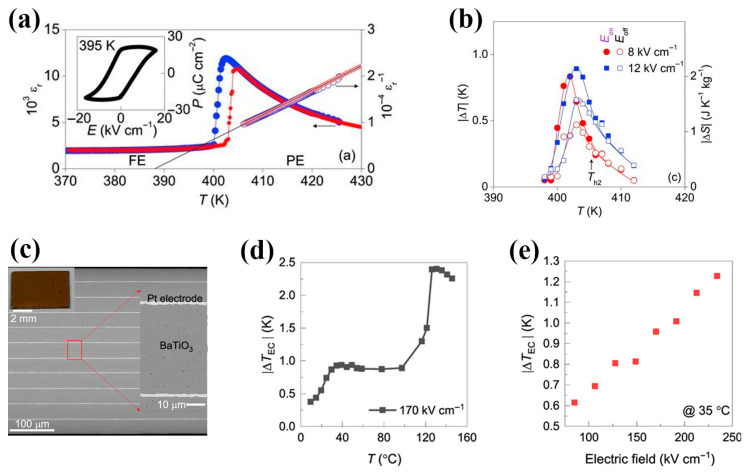
(**a**) Ferroelectric transition in pristine BT single crystal near $T_{C}$. Relative permittivity ($\varepsilon_{r}$) versus temperature T on cooling (blue) and heating (red). A linear fit to the inverse permittivity data above $T_{C}$ ≈ 402 K is also shown (black line). Inset: *P*(*E*) measured just below $T_{C}$. The small high-field reduction is attributed to electrical conductivity at the high measurement temperature. (**b**) Directly measured values of |∆T|, as a function of starting temperature, due to the application (*E*_on_) and subsequent removal (*E*_off_) of 0.8 and 1.2 MV/m [75]. (**c**) Cross-section SEM images of BT MLCC. The inset shows a top view of the MLCC. EC temperature change as a function of (**d**) starting temperature with an excitation of 17 MV/m, and (**e**) electric field at 35 °C. Temperature changes are recorded when the electric field is removed [76].

Intrinsic undoped materials, such as pristine BT, typically exhibit normal ferroelectric (NFE) behaviors. Conventional NFEs undergo a first-order or second-order phase transitions, characterized by the strong dipole–dipole correlations and large spontaneous polarization. However, in the low-temperature ferroelectric phase (far below the Curie point), limited polarization entropy changes when the electric field is removed—due to a large remnant polarization—make it difficult to obtain a large ECE, failing to meet practical cooling demands. Near the Curie point, the first-order phase transition materials display higher pyroelectric coefficients and latent heats, enabling superior ECE within an extremely narrow temperature window. For instance, as shown in Figure 4a,b, Moya et al. [75] reported a 0.8 K adiabatic temperature change in pure BT single crystals near the Curie point under 1.2 MV/m, but the narrow operational range (~3 K) hindered practical applications. In 2023, Li et al. [76] achieved ~0.9 K adiabatic temperature change over ~70 K near the orthorhombic-to-tetragonal phase transition temperature (~30 °C) in BT MLCC under 17 MV/m, and 2.4 K over ~20 K near the Curie point (~126 °C) (see Figure 4c–e). Despite expanded operational ranges (~20 K) at higher fields, these first-order phase transition materials remain unsuitable for broad temperature applications, as their performance is confined to a specific temperature interval.

**Figure 5:**
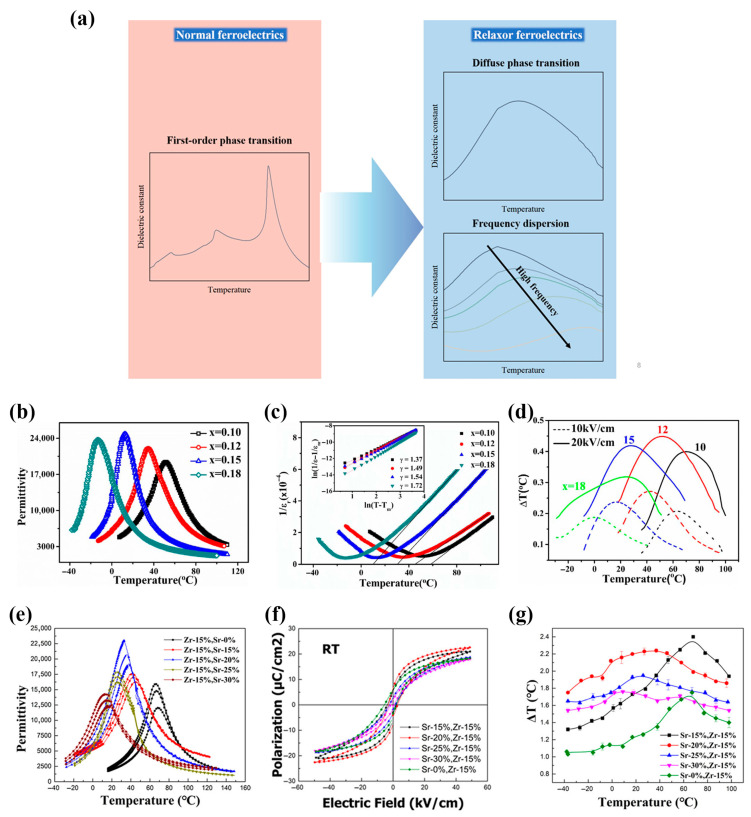
(**a**) Schematic diagram of construction of relaxor ferroelectrics. (**b**) Temperature dependence of dielectric permittivity for Ba(Sn_*x*_Ti_1−*x*_)O_3_ (measured at 10 kHz). (**c**) Temperature dependence of inverse dielectric constant of BST ceramics. In the inset, the double-logarithmic plots deduce diffuseness constant γ. (**d**) Indirect ECE characterization of BST ceramics at selected electric fields. [77] (**e**) Permittivity as a function of temperature for BSZT bulk ceramics measured at 100 Hz, 1 kHz, and 10 kHz: Sr^2+^-doped Ba(Zr_0.15_Ti_0.85_)O_3_; (**f**) polarization as a function of electric field in Sr^2+^-doped Ba(Zr_0.15_Ti_0.85_)O_3_ in RT; (**g**) direct measurement of electrocaloric response as a function of temperature in Sr^2+^-doped Ba(Zr_0.15_Ti_0.85_)O_3_ bulk ceramics under an electric field of 5 MV/m [78].

To address this issue, researchers have modified A- and/or B-sites in perovskite (ABO_3_) structures via doping, inducing compositional and structural fluctuations. It is important to note that the role of doped ions is not to directly induce polar nanoregions (PNRs), but rather to indirectly influence their evolution by suppressing homogeneous strain distortions and the transverse correlation between 1-D PNRs, thereby preventing the transition into a long-range ordered ferroelectric state [109,110]. Therefore, PNRs appear and are conducive to extending the operational temperature window of a large ECE. A schematic diagram of the construction of RFEs is shown in Figure 5a. Researchers have made remarkable progresses by constructing RFEs. For example, with the increase in Sn^4+^ content, the relaxor ferroelectric behaviors of the material are progressively enhanced (Figure 5b,c), leading to a broadening of the EC temperature span (Figure 5d). As a result, a wide-temperature-range electrocaloric effect near room temperature was realized, with a maximum ∆T of ~0.45 K achieved in the Ba(Sn_0.12_Ti_0.88_)O_3_ sample at 2 MV/m and ~57 °C [77]. As shown in Figure 5e–g, Jian et al. reported that a maximal ∆T of 2.43 K was directly measured in (Ba_0.85_Sr_0.15_)(Zr_0.15_Ti_0.85_)O_3_ RFEs at ~68 °C, while a high ECE (>1.6 K) was also maintained over a broad temperature range (120 °C) [78]. These results highlight two advantages of RFEs: (1) ECE over a broad-temperature-range and (2) reduced energy barriers for polarization switching, enabling higher low-electric-field-induced EC responses. Nevertheless, compositional fluctuations weaken the long-range ordering, potentially reducing the polarization and the latent heat during a phase transition, thus diminishing the maximal ECE compared to NFEs with a first-order phase transition. Optimizing the fabrication techniques (e.g., hot-pressing and tape-casting) or adopting advanced architectures (e.g., thick films and MLCCs) to enhance ∆T the breakdown of electric fields could enable RFEs to compensate for reduced polarization at high electric fields [59]. On the other side, increased PNRs may also provide more polarization states, thus enhance the ECE isothermal entropy change ∆S in accordance with the Gibbs–Shannon entropy [111], i.e.,

(20) $$S_{dip}=-\frac{k}{V}\;\sum_{i=1}^{\Omega }N_{i}ln\frac{N_{i}}{N},$$

where *N_i_* is the number of dipoles along the symmetry direction i with the condition $\sum_{i}N_{i}=N$, $N_{i}\;=\;N/\Omega$, *k* is the Boltzmann constant, and *V* the volume of the material. Therefore, the polar states may also contribute to the ECE ∆T and ∆S.

### 5.2. Multi-Phase Coexistence

By constructing a multi-phase coexistence material system, the potential barriers for the reorientation of electric dipoles within the material will be reduced, which means that the flipping and/or reorientation of the electric dipoles becomes easier under the application of an external electric field. Moreover, the multi-phase coexistence can lead to more possible polarization components, which may enhance the ∆S. This is expected to enhance the ferroelectric polar properties as well as the ECE of the material. Currently, the construction of a multi-phase coexistence system is mainly achieved via the phase-boundary control. Specifically, it involves constructing the phase boundaries and the critical points at the triple critical points of two or more ferroelectric phases to improve their physical properties in terms of the Landau–Devonshire free energy expression. Conventional examples include the morphotropic phase boundary (MPB) [112,113,114,115] and the invariant critical point (ICP) [80,116,117,118,119]. Considering the influencing factors of various phase boundaries, the MPB has great temperature stability and is only related to the material composition, which is not affected by the temperature; the ICP has a stronger temperature dependence and is also affected by the material composition. Its advantage lies in the ability to construct more possible phase compositions.

MPB commonly appears in the binary systems, e.g., (1−*x*)PbZrO_3_-*x*PbTiO_3_ [112], (1−*x*)Pb(Mg_1/3_Nb_2/3_)O_3_ -*x*PbTiO_3_ [113], and BT-based solid solutions with low BT contents, such as (1−*x*)Na_0.5_Bi_0.5_TiO_3_-*x*BaTiO_3_ [114]. It is rarely observed in solid solutions with a high BT content and will therefore not be discussed here in detail.

**Figure 6:**
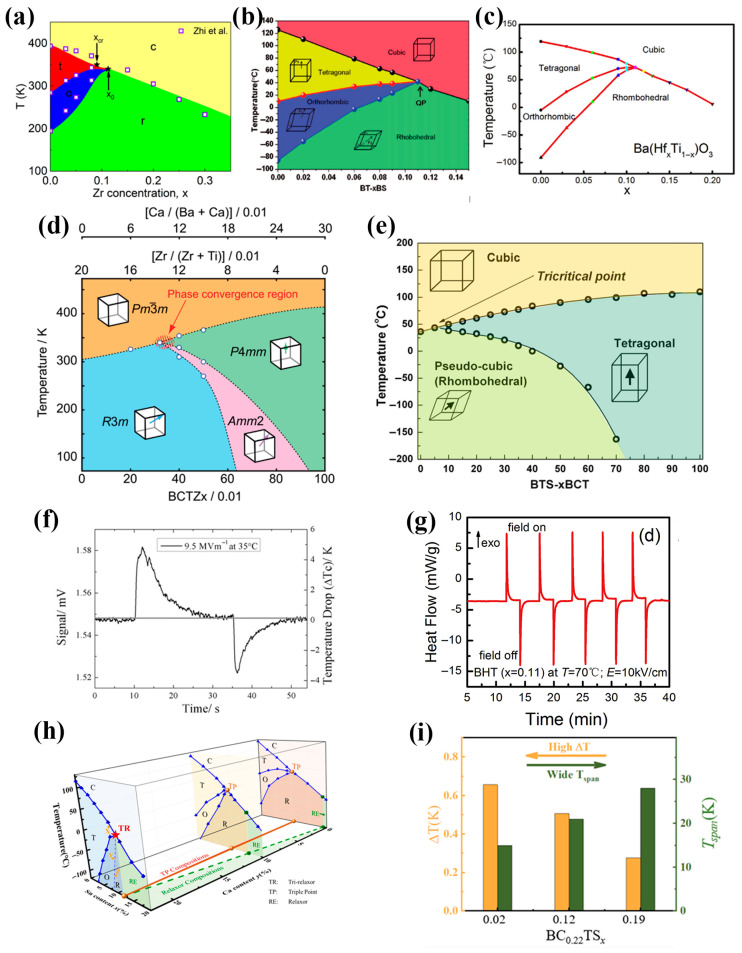
Temperature–composition phase diagram of typical material systems with ICP. (**a**) Ba(Zr_*x*_Ti_1−*x*_)O_3_ [116]; (**b**) Ba(Sn_*x*_Ti_1−*x*_)O_3_ [117]; (**c**) Ba(Hf_*x*_Ti_1−*x*_)O_3_ [80]; (**d**) Ba(Ti_0.8_Zr_0.2_)O_3_-(Ba_0.7_Ca_0.3_)TiO_3_ [118]; (**e**) Ba(Sn_0.12_Ti_0.88_)O_3_-*x*(Ba_0.7_Ca_0.3_)O_3_ [119]; (**f**) the directly recorded ECE signal for BZT(*x* = 0.2) as the electric field was turning on and off, respectively. The data were measured under Δ*E* = 9.5 MV/m at 35 °C. Solid line was drawn to show the ambient temperature reading [79]. (**g**) The direct measurement of the BHT ceramic with *x* = 0.11 [80]. (**h**) Phase diagrams of the (Ba_1−*y*_Ca*_y_*)(Ti_1−*x*_Sn*_x_*)O_3_ system (abbreviated as BC*_y_*TS*_x_*, 0 ≤ *x* ≤ 0.2, *y* = 0, 0.1, 0.22), denoting the corresponding triple point (TP) and relaxor region (RE). TR (at ∼20 °C, as shown by red star) is induced in a triple point–relaxor crossover at BC_0.22_TS_0.12_ (featured by a mixture of tetragonal (T), orthorhombic (O), and rhombohedral (R) PNRs. (**i**) Comparison of the adiabatic temperature change ∆T and temperature stability *T*_span_ for BC_0.22_TS*_x_* materials [81].

Typical material systems with ICP mainly concentrate on BT-based systems. By doping various ions to achieve the permittivity “peak-shifting effect” between the ferroelectric phases and the ferroelectric phases or the paraelectric phase, three-phase or four-phase critical points might be constructed. Figure 6a–e shows phase diagrams of the material systems obtained by doping Zr^4+^, Sn^4+^, Hf^4+^, and Ca^2+^ ions alone or two of them together in BT [80,116,117,118,119] Clearly, ICP is sensitive to the material system and particularly to the temperature. The shift of composition may lead to a significant attenuation of performance. Based on the definition of the ECE, when the external electric field changes, the greater the change in the polarization entropy of the electric dipoles, the larger the ECE. If the polarization disorder of the material is improved through the coexistence of multiple phases, that is, by having multiple polarization states (more spontaneous polarization orientations), the polarization entropy of the electric dipoles of the material system can be significantly increased. Liu et al. [120] proposed a theoretical framework based on thermodynamics to describe the expression of the dipole entropy (*S_dip_*) of the system under ideal conditions in order to better explain the origin of the high ECE of the material system at the ICP. Its expression is shown in Equation (21):

(21) $$S_{dip}=-\sum_{i}\frac{k}{v_{i}}c_{i}ln(c_{i}/\Omega_{i}),$$

where *k* is the Boltzmann constant (1.380649 × 10^−23^ J/K), $c_{i}$ is the volume fraction of the *i*-th phase, $\Omega_{i}$ denotes the number of polarization states in the *i*-th phase, and $v_{i}$ represents the volume of each polar unit. For instance, for the material system with the R phase, there are 8 equivalent polarization orientations along the <111>_c_ directions; for the material system with the O phase, there are 12 equivalent polarization orientations along the <110>_c_ directions; for the material system with the T phase, there are 6 equivalent polarization orientations along the <100>_c_ directions [121]. In addition, the monoclinic phases (M phase) also have eight equivalent polarization orientations. Taking the multi-phase coexistence system as an example, the R-T coexistence material system has 14 possible polarization orientations, and the R-O-T coexistence material system has 26 possible polarization orientations, etc. Clearly, the coexistence of multiple phases in the material system can significantly increase the possible polarization orientations and thereby increase the initial dipole entropy of the material, which is conducive to the improvement in the ECE.

Based on the “multi-phase coexistence” strategy, researchers have already explored novel EC material systems. Qian et al. [79] prepared Ba(Zr_0.2_Ti_0.8_)O_3_ ferroelectric ceramic, which is close to the ICP of multi-phases (i.e., C phase, T phase, R phase, and O phase) through the conventional solid-state reaction method, achieving a giant ECE near room temperature. At 14.5 MV/m and 39 °C, the ∆T value of 4.5 K was obtained (see Figure 6f). Moreover, Li et al. [80] explored the dielectric properties, ferroelectric properties, and ECE of ceramics with different Hf^4+^ contents doped in BT ceramics. By comparing the ECE of materials with a lower doping (NFE state), at the ICP (multiphase coexistence state), with those with a higher doping (RFE state), the best ECE was obtained in the ICP composition Ba(Hf_0.11_Ti_0.89_)O_3_ coexisting with the R phase, O phase, T phase, and the C phase. As shown in Figure 6g, the maximum ∆T of 1.35 K was obtained under 5 MV/m. Meanwhile, thanks to the reduction in the coercive field, the ECE of the material was significantly improved at low electric fields, mainly manifested with a larger EC strength value of 0.35 K/(MV·m) at 1 MV/m for ceramics. Although the material compositions located in the ICP achieved relatively better ECE, when the doping amount was not high, the RFE properties were not strong, which means that these better ECE properties only existed in a slightly wider temperature range than those of the NFE. Based on this, Wang et al. [81] designed a tri-RFE phase in (Ba_1−*y*_Ca_*y*_)(Ti_1−*x*_Sn_*x*_)O_3_ via the compositional tailoring (see Figure 6h,i), achieving a high dielectric constant (~18,000) in a wide temperature range (34 K). Due to the existence of PNRs in the R phase, O phase, and T phase, the (Ba_0.78_Ca_0.22_)(Ti_0.88_Sn_0.12_)O_3_ exhibited a ∆T of 0.5 K at 2.5 MV/m, as well as excellent ECE performance over a temperature range of 21 K.

**Figure 7:**
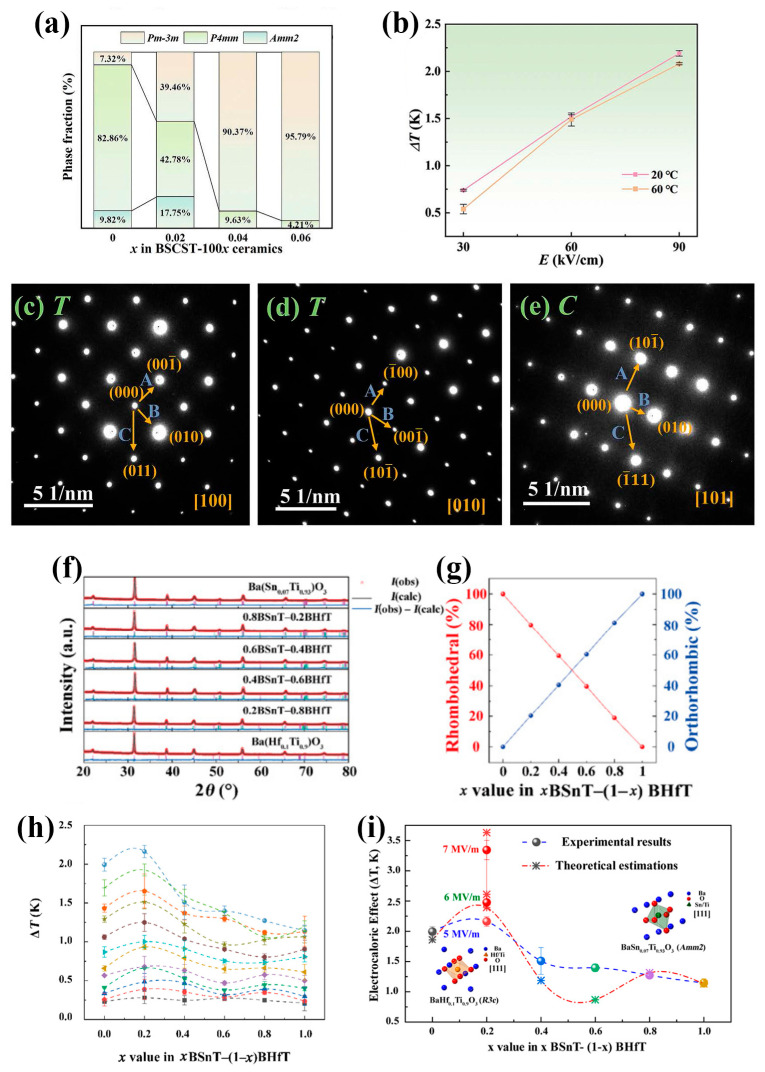
(**a**) Tetragonal, orthorhombic, and cubic phase fraction in (Ba_0.65_Sr_0.3_Ca_0.05_)(Sn_x_Ti_1−x_)O_3_ (BSCST-100*x*) ceramics (*x* = 0, 0.02, 0.04, and 0.06). (**b**) Δ*T* of BSCST-2 ceramics calculated from heat flow. (**c**–**e**) SAED patterns along the zone axis (*x* = 0) [1,0,0] and (*x* = 0.02) [0,1,0], [1,0,1], corresponding to the T and C phase regions [37]. (**f**) XRD patterns of *x*Ba(Sn_0.07_Ti_0.93_)O_3_–(1−*x*)Ba(Hf_0.1_Ti_0.9_)O_3_ (*x*BSnT–(1−*x*)BHfT) ceramics. (**g**) Phase fractions of orthorhombic phase (blue line) and rhombohedral phase (red line) related to different compositions. (**h**) Directly measured ECE results for *x*BSnT–(1−*x*)BHfT ceramics under 5 MV/m. (**i**) Directly measured ECE results for *x*BSnT–(1−*x*)BHfT ceramics under different electric fields [18].

In addition to tailoring the material compositions to ICP to enhance the ECE, researchers have also focused on the construction of multi-phase coexistence as an effective way to improve the ECE. For instance, as shown in Figure 7a–e, Lin et al. [37] introduced Sn^4+^ ions into (Ba_0.65_Sr_0.3_Ca_0.05_)TiO_3_, resulting in the formation of a multi-phase structure. The optimized composition, (Ba_0.65_Sr_0.3_Ca_0.05_)(Sn_0.02_Ti_0.98_)O_3_, exhibited the highest ∆T of 1.79 K (indirect) and 2.18 K (direct) at 20 °C. Specifically, multi-phase coexistence increased the number of ferroelectric domains—thereby boosting the *S_dip_*—and simultaneously flattened the free energy profile, which facilitated easier polarization rotation under an applied electric field. Moreover, as shown in Figure 7f–i, Niu et al. synthesized *x*Ba(Sn_0.07_Ti_0.93_)O_3_–(1−*x*)Ba(Hf_0.1_Ti_0.9_)O_3_ (xBSnT−(1−x)BHfT) ceramics via a traditional solid-state reaction process by separately doping BaTiO_3_ with Sn^4+^ and Hf^4+^ ions. They proposed a “polarization merging” model, in which the dielectric response of the coexisting phases was not a linear superposition of the individual dielectric behaviors. Instead, electric-field-induced polarization merging between the two phases occurred, providing a reasonable explanation for the superior ECE properties observed in the systems located near the MPB or the ICP. Finally, optimal ECE was achieved in 0.2BSnT–0.8BHfT ceramics, showing a $\Delta T_{max}$ of 3.35 ± 0.09 K at 80 °C under an electric field of 7 MV/m [18]. It can be seen that developing and improving systems with multi-phase coexistence are obviously of great significance for developing materials systems with high ECEs.

### 5.3. Control of Oxygen Vacancies

During high-temperature sintering, the formation of oxygen vacancies is almost inevitable. The concentration of oxygen vacancies directly affects the electrical properties of ferroelectric ceramics, including the impedance, dielectric loss, fatigue resistance, etc. In BT-based ceramics, excessive oxygen vacancies can significantly increase the leakage current, degrade the insulation, and even lead to semiconductor-like electric behaviors. Therefore, effectively controlling the generation and concentration of oxygen vacancies is critical for optimizing the electrical and ECE performance of the materials. At present, three main strategies have been proposed by researchers to regulate oxygen vacancies.

#### 5.3.1. Compensation of Oxygen Vacancies via Doping with Multivalent Ions with a High Valence

**Figure 8:**
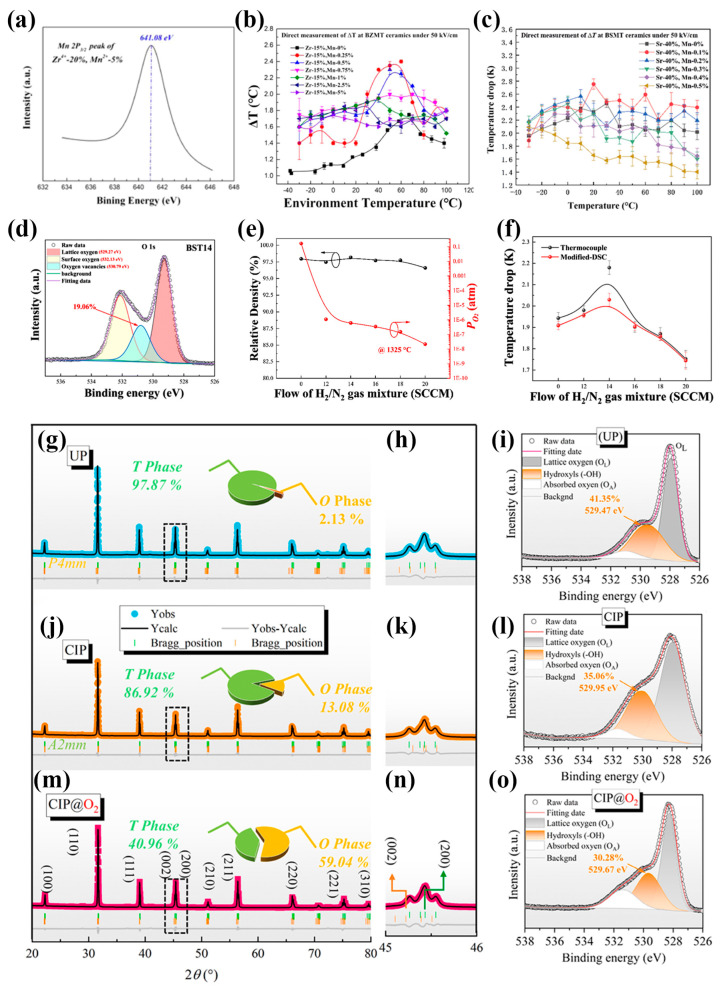
(**a**) XPS spectra of Mn 2p3/2 peaks in Ba(Zr*_x_*Mn*_y_*Ti_1−*x*−*y*_)O_3_ samples, where *x =* 0.20 with *y* = 0.05; (**b**) ΔT as a function of temperature in Zr^4+^, Mn^2+^-codoped BT ceramics under 5 MV/m [82]. (**c**) ΔT for Mn^2+^-doped BST ceramics directly measured at temperatures ranging from –30 to 100 °C under 5 MV/m: Sr = 40% [83]. (**d**) X-ray photoelectron spectra of the oxygen element in Ba_0.7_Sr_0.3_TiO_3_ bulks sintered under different oxygen partial pressures. (**e**) Relative density of Ba_0.7_Sr_0.3_TiO_3_ bulks and specific value of oxygen partial pressure as a function of flow rate of the H_2_/N_2_ gas mixture at 1325 °C. (**f**) Comparison of DSC and thermocouple-derived ECE results [84]. (**g**,**j**,**m**) XRD patterns of the ceramics for different forming methods. Additionally, the zoomed-in profile within dashed squares in the range of 2*θ* = 45–46.0 are shown in panels (**h**,**k**,**n**). (**i**,**l**,**o**) X-ray photoelectron spectra of oxygen in BSZMT ceramics with different forming methods [38].

Doping with appropriate amounts of multivalent ions with a high valence, such as Mn^4+^, has been proven effective in compensating for the oxygen vacancies generated during sintering, thereby improving the dielectric, ferroelectric, and ECE properties of BT-based ceramics. At low concentrations, Mn^4+^ ions can form electrically neutral defect pairs with oxygen vacancies, which suppress free carrier generation and migration, reduce leakage current, and enhance insulation resistance. However, as the Mn^4+^ concentration increases further, two adverse effects may occur: (i) Mn^4+^ ions, acting as acceptor-type defects, may introduce additional carriers and trap centers, leading to a higher leakage current and deteriorated performance; (ii) these defects may form immobile dipole complexes, hindering the field-induced switching of the neighboring dipoles, thus weakening the material’s polarization response and the ECE. Therefore, the Mn^4+^ doping level must be precisely optimized to achieve a balance between the oxygen vacancy compensation and the improved functional performance. Li [82] and Niu [83] et al. systematically investigated the role of Mn^4+^ doping and established optimal concentration ranges. As shown in Figure 8a,b, Li et al. reported a maximum ΔT of 2.35 K at 62 °C in Ba(Zr_0.15_Mn_0.0025_Ti_0.8475_)O_3_ ceramics, along with a broad operational temperature window of approximately 40 °C. Afterwards, Niu et al. chose typical (BaSr)TiO_3_ and demonstrated a ΔT of 2.75 K with a high EC strength of 0.55 K/(MV·m) in Ba_0.6_Sr_0.4_Mn_0.001_Ti_0.999_O_3_ relaxor ferroelectric ceramics near room temperature (see Figure 8c), with stable EC responses (ECE ΔT > 2.2 K) maintained over a wide temperature range (~120 K), indicating promising application potential.

#### 5.3.2. Regulation of Oxygen Partial Pressure During Sintering

Controlling the oxygen partial pressure (*P*_O2_) during sintering is a critical issue in manipulating the formation of oxygen vacancies. In a reduced atmosphere, excessive oxygen vacancies may be formed, degrading the material’s electrical insulation. As shown in Figure 8d–f, Liang et al. [84] investigated the Ba_0.7_Sr_0.3_TiO_3_ (BST) ceramics with a phase transition near room temperature, and a H_2_/N_2_ gas mixture was used to tune the (*P*_O2_) during the sintering. Their findings revealed that at a *P*_O2_ of 6.14 × 10^−7^ atm, Ni electrodes remained unoxidized, while the oxygen vacancy concentration was effectively suppressed. Under these conditions, the material exhibited a ∆T of 2.18 K at 40 °C under a 4 MV/m electric field, confirming the feasibility of oxygen atmosphere engineering for ECE enhancement.

#### 5.3.3. Post-Sintering Treatment to Optimize the Oxygen Vacancy Concentration and Microstructure

Post-sintering treatments, especially those involving oxygen-rich environments, can further adjust the oxygen vacancy concentrations and refine the microstructure and phase composition of the ceramics. As shown in Figure 8g–o, recent studies have shown that different forming methods, such as annealing in an oxygen atmosphere, improve the densification, decline the defect concentration, and help stabilize the multi-phase coexistence. For example, in Ba(Sr,Zr,Ti)O_3_ ceramics, oxygen annealing processing resulted in a balanced coexistence of tetragonal and orthorhombic phases. Moreover, moderate levels of oxygen vacancies facilitate the formation of PNRs, enhancing the ferroelectric response and ECE. As a result, these ceramics exhibited robust EC responses (ΔT > 0.5 K) over a wide temperature window from 15 °C to 100 °C, with a peak ΔT of 1.30 K at 70 °C under a lower electric field [38].

### 5.4. High-Entropy Design

In accordance with the definition of ECE, the change in polarization entropy ($∆S_{polar}$) within polar materials caused by an electric field leads to the ECE. Thus, it is expected that constructing EC materials with a high $∆S_{polar}$ can achieve a large ECE.

**Figure 9:**
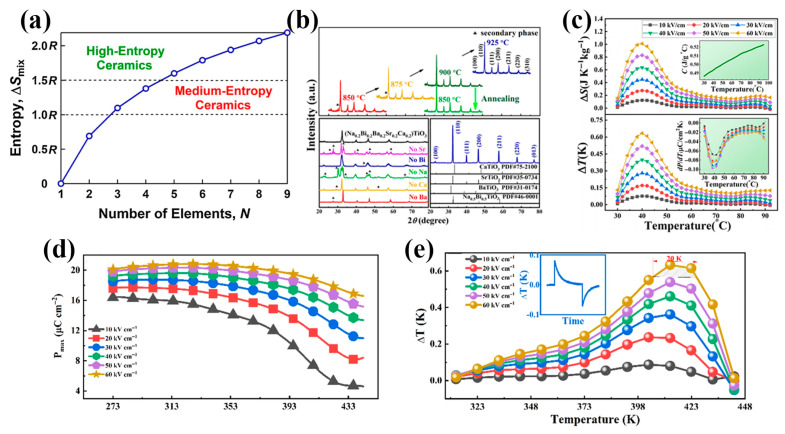
(**a**) Relation between entropy of mixing and number of elements and definition of medium-entropy ceramics and high-entropy ceramics [124]. (**b**) XRD patterns of (Na_0.2_Bi_0.2_Ba_0.2_Sr_0.2_Ca_0.2_)TiO_3_ (NBBSCT) powders calcined at different temperatures, a composition series where individual components are removed from the parent composition NBBSCT. (**c**) ΔS and ΔT are functions of temperature for the NBBSCT ceramic at different electric fields. The inset shows the specific heat capacity and pyroelectric coefficient as a function of temperature under different electric fields [85]. (**d**) *P*_max_ as a function of temperature under different electric fields for (Bi_1/6_Na_1/6_Sr_1/6_Ba_1/6_Pb_1/6_Ca_1/6_)TiO_3_ (BNSBPC) ceramic. (**e**) ΔT as a function of temperature under different electric fields for BNSBPC ceramic. The inset of (**e**) shows the ΔT of BNSBPC measured using a direct method under 6 MV/m at room temperature [86].

The concept of high entropy originated from the high-entropy alloys. In 2004, a new type of multi-component high-entropy alloy emerged in the field of metallic materials, breaking through the limitations of traditional main compositional design of alloys [122]. Since then, high-entropy materials have become a research hotspot for researchers worldwide. Here, the entropy mainly refers to the configurational entropy ($∆S_{config}$). The essence of “high entropy” in high-entropy materials is that their $∆S_{config}$ is greater than 1.5R (R is the gas constant, R = 8.314 J/(mol·K)). In general, materials formed by five or more chemical elements with similar or equal molar ratios have a $∆S_{config}$ exceeding 1.5R, thus forming a high-entropy material (see Figure 9). For high-entropy ceramics with a perovskite structure, considering the existence of A-site doping and B-site doping, the general calculation formula for $∆S_{config}$ is [123]

(22) $$∆S_{config}=-R[{\left(\sum_{i=1}^{n}x_{i}lnx_{i}\right)}_{cation-site}+{\left(\sum_{j=1}^{n}x_{j}lnx_{j}\right)}_{anion-site}],$$

where $x_{i}$ and $x_{j}$ represent the molar ratios of elements at cationic and anionic sites, respectively. In recently published papers, $∆S_{config}\leq 1.0R$ is classified as low-entropy ceramics, $1.0R\leq ∆S_{config}\leq 1.5R$ as medium-entropy ceramics, and $∆S_{config}\geq 1.5R$ as high-entropy ceramics.

Thanks to the different influences of different elements on the phase compositions and microstructures of materials, the cocktail effect introduced by the “high-entropy design” strategy can lead to unexpected performance. In addition, in the original doping strategy, elements with similar electronegativities, the same valences, and similar ionic radii to the original ions in the perovskite structures are often selected for doping. However, the “high-entropy design” strategy, which involves doping multiple elements at the same site, is bound to introduce more types of lattice distortions. Combined with the coupling effects between different elements, it may even introduce special domain structures, more phase compositions, and more types of oxygen octahedral distortions. Therefore, the “high-entropy design” strategy is expected to achieve ceramic material systems with more excellent functional properties.

At present, the design mechanism of high-entropy materials is not yet clear. Researchers in China and abroad mainly focus on increasing the $∆S_{config}$ of materials when designing high-entropy ferroelectrics. Pu et al. [85] successfully prepared a five-element equal-molar substituted high-entropy ceramic Na_0.2_Bi_0.2_Ba_0.2_Sr_0.2_Ca_0.2_TiO_3_ ($∆S_{config}=1.61R$). Through the calculation of thermodynamic parameters, it was found that both the entropy and the enthalpy jointly drive the formation of a stable system. Meanwhile, XRD results indicated that it was a stable pseudo-cubic phase (Pm$\bar{3}$m point group). Based on the Maxwell relation, the optimal ECE of 0.63 K was obtained under an applied electric field of 6 MV/m. Liu et al. [86] reported two high-entropy ceramics with equal six-element substitution, namely, (Bi_1/6_Na_1/6_Sr_1/6_Ba_1/6_Pb_1/6_Ca_1/6_)TiO_3_ and (Bi_1/6_La_1/6_Na_1/6_K_1/6_Sr_1/6_Ba_1/6_)TiO_3_. They measured the ECE of both materials at different temperatures and electric fields using the direct measurement method. The results showed that the former was a single T phase (P4mm space group), obtaining a relatively stable ECE in a narrow temperature range (~20 K), with a maximum adiabatic temperature change of 0.63 K at 6 MV/m. The latter was a single pseudo-cubic phase (Pm$\bar{3}$m space group), obtaining a relatively stable ECE in a wide temperature range (100 K), with a maximum ∆T of 0.14 K. The above researchers merely explored the “high-entropy design” based on the design of high $∆S_{config}$, providing many inspirations for the “high-entropy design”. By increasing the configurational entropy to design high-entropy ceramics, the RFE properties and temperature stability will be greatly enhanced. However, due to the high substitution of multiple ions at the A or B sites, fluctuations in composition and structure will inevitably cause the material to be closer to a pseudo-cubic structure, resulting in a significant reduction in saturation polarization and a possible decrease in the ECE. Therefore, it is still a major challenge to achieve high ECEs in inorganic materials using the “high-entropy strategy”.

From the perspective of improving the ECEs of materials, the high-entropy design should focus on increasing the initial $∆S_{polar}$ of the material rather than only considering the $∆S_{config}$. As mentioned earlier, the traditional way to increase the initial polarization entropy of materials is to construct phase boundaries such as MPB and ICP, increasing the number of possible polarization orientations and increasing the initial orientation disorder of polarization within the material. Relevant examples have been described in detail in the “Multi-phase Coexistence” section. Currently, new ways to increase the initial polarization entropy are still under exploration. In the meantime, alternative methods to boost the initial polarization entropy are still being actively explored. This increase in initial entropy resembles that achieved through “Multi-phase Coexistence” in local structure, providing a new empirical pathway for designing high-entropy polar ceramics. Nonetheless, the fundamental mechanisms underlying the relationship between $∆S_{polar}$ and $∆S_{config}$ remain insufficiently understood and warrant further theoretical and experimental investigations.

### 5.5. Improving the Dielectric Breakdown Strength (DBS)

According to the Maxwell relation and Landau phenomenological theory, the ECE is positively correlated with the *E*. At low electric fields, $∆T\propto E^{2}$, and at higher electric fields, $∆T\propto E^{\frac{2}{3}}$ [25]. It can be seen that the improvement in the DBS is also a key issue in enhancing the ECE. Meanwhile, in practical application scenarios, to ensure the reliability of working conditions/devices and extend the operation life, the materials are often used at 20–50% of their DBS. Therefore, improving the DBS is also a critical way to increase the lifetime of the ECE cooling devices.

Currently, the mainstream strategies to enhance the DBS of ferroelectric ceramics fall into two major categories: compositional control and structural control. Compositional control includes bandgap engineering [87], grain size engineering [125], and grain boundary modification (e.g., glass phase addition or core–shell design) [88]. Structural control primarily refers to macroscopic architectural design—such as rolling, tape casting, or multilayer stacking—to fabricate thick-film ceramics or MLCCs, thereby reducing the thickness of a single ceramic layer and mitigating the inhomogeneities of both the microstructure and the electric field [76].

**Figure 10:**
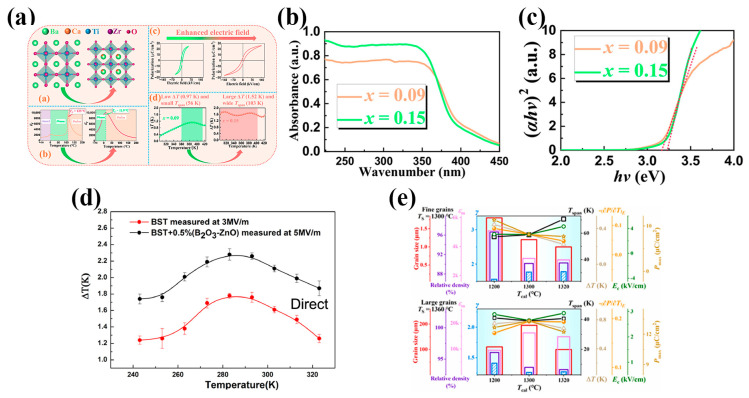
(**a**) Crystal structures of components; temperature-dependent dielectric constants of pure BT and doped BT; ferroelectric hysteresis loops for 9 and 15 mol% CaZrO_3_-doped BT; temperature dependence of ∆T for 9 and 15 mol% CaZrO_3_-doped BT. (**b**,**c**) UV–Vis spectra and (*αhv*)^2^–*hv* curves for *x* = 0.09 and *x* = 0.15 ceramic samples [87]. (**d**) Ba_0.65_Sr_0.35_TiO_3_ and Ba_0.65_Sr_0.35_TiO_3_ + 0.5 wt% (B_2_O_3_-ZnO) ceramics: ∆T as a function of temperature by direct measurement [88]. (**e**) Evolution of grain size, relative density, *ε*_m_ (at 1 MHz), *γ*, *E*_c_, *P*_max_ (4 MV/m), peak (∂*P*/∂*T*)*_E_*, $∆T_{\max}$ and *T*_span_ (4 MV/m) for fine-grain and large-grain BZT0.15 ceramic at different *T*_cal_ [125].

As shown in Figure 10a–c, Wang et al. [87] introduced a high-bandgap CaZrO_3_ into BT-based ceramics, which effectively increased the breakdown field. As a result, they successfully achieved a ∆T of 1.78 K and a broad operational temperature span of 103 K (∆T > 1.52 K) near room temperature. This demonstrates the potential of bandgap engineering as a means of simultaneously enhancing both the DBS and the ECE in lead-free ferroelectric materials. The DBS *E*_b_ can be categorized as intrinsic or extrinsic ones. Intrinsic *E*_b_ is controlled primarily by the electronic band gap (*E*_g_), chemical bonds, and crystal-field effects, while extrinsic *E*_b_ comes from the microstructural, interface, electric field distribution, etc., factors (e.g., pores, vacancies/defects, grain boundaries, and interface between the ceramic layer and the electrode, etc.). *E*_g_ denotes the energy required for electron excitation from the valence band to the conduction band and is often found to correlate empirically with the intrinsic *E*_b_ [126]. A larger *E*_g_ means that more energy is required for electrons to transition from the valence band to the conduction band, making it more difficult for electrons to be excited as free charge carriers. Therefore, under an applied electric field, materials with a larger *E*_g_ generally exhibit higher thresholds for electric breakdown. The *E*_g_ can be evaluated from the ultraviolet–visible (UV–vis) absorption spectroscopy by considering an indirect or direct electronic transition, depending on the material. For indirect ones (most cases), the Tauc equation of (*αhν*)^2^ ~ (*hν*-*E*_g_) (*α* is the absorbance, *ν* is the optical frequency, and *h* is the Planck constant) was applied.

For extrinsic breakdown, a Griffith-type model is frequently employed to describe dielectric breakdown. Within this model, the *E*_b_ is mainly determined by both the $\varepsilon_{r}$ and the sample thickness (d), and can be expressed as [126]

(23) $$E_{b}=\frac{1}{c}\sqrt{\frac{6A}{5a_{bd}\pi }\frac{1}{\varepsilon_{r}\varepsilon_{0}}\frac{1}{d}},$$

where *c* is a coefficient, *A* is the material constant, *a_bd_* is the length of conducting filament, and $\varepsilon_{0}$ is the vacuum permittivity. Equation (23) indicates that, for specimens of identical thickness, *E*_b_ is inversely related to $\sqrt{\varepsilon_{r}}$.

Besides grain boundary, modification by introducing a small amount of glass phase has proven effective in enhancing the DBS. However, this approach often introduces weakly polar or non-polar phases into the system, which also reduce the polarization and compromise the ECE under low electric fields. Therefore, a critical challenge in this strategy is how to balance the trade-off between the increased DBS and decreased polarization. Studies have shown that the optimal content of the glass phase should typically lie within 0.1 wt–1 wt%, although the exact value depends on the ceramic and glass compositions. A notable example was reported by Wang et al. [88], where the addition of 0.5 wt% B_2_O_3_-ZnO glass phase to Ba_0.65_Sr_0.35_TiO_3_ bulk ceramics increased the DBS from 3 MV/m to 5 MV/m and simultaneously enhanced the ∆T from 1.70 K to 2.29 K, demonstrating a synergistic improvement in both DBS and ECE (see Figure 10d).

Another important compositional issue is grain size control. As shown in Figure 10e, Xu et al. [125] systematically investigated the relationship between the calcination temperature, sintering temperature, microstructure (i.e., grain size), and the ECE properties. Their results revealed that, under the same applied field, the ceramics with larger grain sizes tend to exhibit stronger ECEs due to easier polarization rotation, higher remanent polarization, and larger polarization variation under the electric field. However, since the grain size is generally inversely correlated with the DBS, achieving a high ECE requires balancing the enhanced polarization of large grains with the higher DBS of ceramic with fine grains. This trade-off remains an important consideration in material design.

Macroscopic structure control is also a relatively effective means to enhance the DBS and to improve the ECE at present. In inorganic ferroelectric materials, due to the DBS being less than 10 MV/m for bulk ceramics, the ECE of inorganic ceramic materials is often lower than that of organic polymers (DBS > 500 MV/m). In practical application situations, to ensure stability, the applied electric field is often lower, making it difficult to fully utilize the inherent excellent ECE. Through the tape-casting method or rolling processes, single-layer thick film ceramics with a smaller thickness can be prepared. Due to the reduction in defect concentration, the DBS gradually approaches the intrinsic DBS^118^, resulting in a significant improvement in the ECE. In addition, considering the low thermal mass of single-layer thick film ceramics, a multi-layer ceramic process can be used to stack single layers to form an MLCC structure for refrigeration units. On the one hand, MLCC increases the thermal mass of the material. On the other hand, MLCC can provide a higher DBS compared to bulk ceramics. In 2014, Ye et al. [89] prepared Ba(Zr_0.2_Ti_0.8_)O_3_ thick films through tape casting and measured a giant ΔT of ~7 K at 40 °C and 19.5 MV/m measured using a modified-DSC, which is much greater than the ECE of the same composition bulk ceramics (4.5 K @ 14.5 MV/m, 39 °C, and 100 μm in thickness [79]). This is attributed to the further enhancement in the DBS due to the reduction in the thickness of a single ceramic layer (~12 μm [89]).

### 5.6. Polarization Flip

The polarization flip strategy was first proposed by Lu et al. [127] in a Pb(Mg_1/3_Nb_2/3_O_3_)-PbTiO_3_ (PMN-PT) single crystal. In NFE, when the electric field is removed, macroscopic domains cannot fully return to their original state due to the large polarization reversal energy barrier, resulting in a certain memory effect and a relatively large remanent polarization. The current electric field excitation method for ECE measurement is mainly the unidirectional square wave electric field excitation method. By applying a unidirectional square wave electric field to the EC material, the dipoles are all reoriented along the electric field direction for a period of time before the electric field is removed (0→+*E*→0). When a unidirectional electric field is applied to the EC material with NFE properties, it has a large polarization change ($\Delta P=P_{\max}$), and there is a large heating peak at this time. However, when the electric field is removed, there is only a very small polarization change ($\Delta P=P_{\max}-P_{r}$), corresponding to a small ΔS and ΔT. Using this electric field excitation method, it is difficult to fully utilize the advantage of the significant saturation polarization of NFE materials.

**Figure 11:**
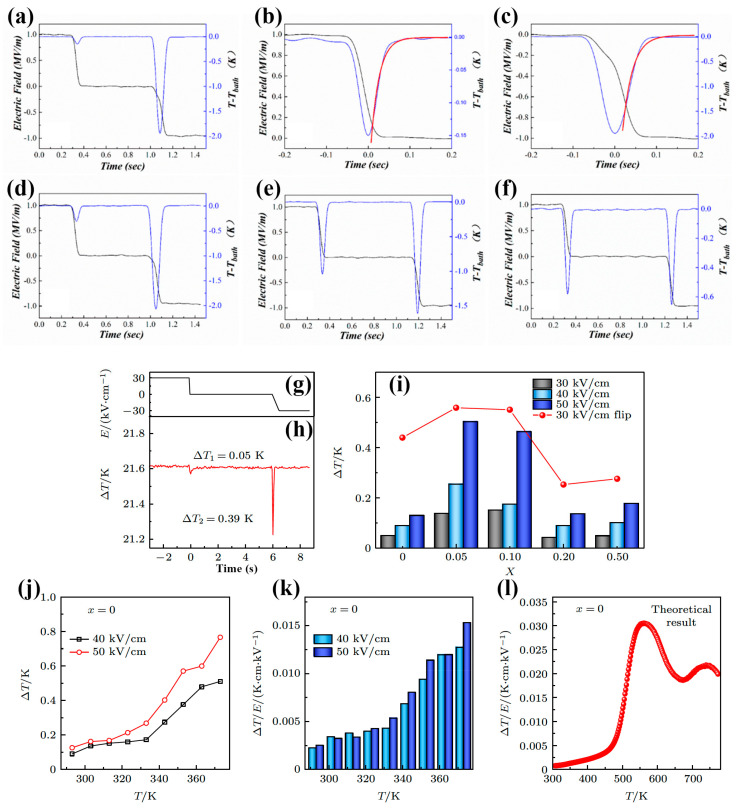
The ECE signals of [110] direction of PMN-PT single crystal at 1 MV/m [127]. (**a**) ECE measured @ 296 K; (**b**) fitting of 1st signal in (**a**); (**c**) fitting of 2nd signal in (**a**); (**d**–**f**) ECE measured @ 353, 383, and 418 K, respectively. (**g**) The change of electric field when the ECE of 0.7BiFeO_3_-0.3BaTiO_3_ (BFO-BTO) + 0% MnO_2_ (Mn0) ceramics measured [90]; (**h**) the direct measurement electrocaloric ΔT of Mn0 ceramics during the electric field changes from +3 MV/m to 0 MV/m and 0 MV/m to −3 MV/m; (**i**) the ΔT of BFO-BTO + *x*% MnO_2_ ceramics at different electric field and the ΔT of BFO-BTO + *x*% MnO_2_ ceramics with polarization flip during the electric field changes from +3 MV/m to −3 MV/m; (**j**) the ΔT of BFO-BTO ceramics at different temperatures under 4 MV/m and 5 MV/m; (**k**) the ΔT/ΔE of Mn0 ceramics at different temperatures under 4 MV/m and 5 MV/m; (**l**) the theoretical ΔT/ΔE of Mn0 ceramics.

Lu et al. applied an electric field of 1 MV/m to the PMN-PT sample and maintained it for a few seconds to reach temperature equilibrium first. Then, the electric field was removed immediately. Meanwhile, the first ECE signal appeared. After the temperature equilibration, a reversed electric field of 1 MV/m (−1 MV/m) was applied to the sample. Then, the second ECE signal can be detected. Furthermore, during the same test run, it can also be noticed that the intensity of the first ECE signal is over 10 times smaller than that of the second ECE signal at low temperature, but the intensity difference of the two ECE signals becomes narrower with increasing temperature. Figure 11a,d–f illustrates the ECE signals of [110] PMN-PT at 296 K, 353 K, 383 K, and 418 K, respectively. In general, before fitting, by making use of the transformation of coordinate translation, the peak value was set to zero, as described in Figure 11b,c. The red curves are the fitted curves. ΔT was obtained by extrapolating the fitting toward the time of the fall of the step-like pulse. In the direct measurement of ΔT, one concern is the Joule heating in the samples, which causes a lowering of temperatures when the field is removed. However, during the test, the baseline temperature *T*-*T*_bath_ in Figure 11 remains the same except for the application of or withdrawal of the electric field, which indicates that the observed temperature change is indeed due to the ECE from the crystals.

The corresponding ΔT and ΔS during this electric field excitation process can be expressed as follows:

(24) $$0\to +E:\;∆P=P_{\max},\;∆S=-\frac{1}{2}\beta \left(P_{m}^{2}\right);$$

(25) $$+E\to 0:\;∆P=P_{\max}-P_{r},\;∆S=-\frac{1}{2}\beta \left(P_{r}^{2}-P_{m}^{2}\right);$$

(26) $$0\to -E:\;∆P=P_{\max}+P_{r},\;∆S=-\frac{1}{2}\beta {\left(P_{r}+P_{m}\right)}^{2};$$

(27) $$-E\to 0:\;\Delta P=P_{\max}-P_{r},\;∆S=-\frac{1}{2}\beta \left(P_{m}^{2}-P_{r}^{2}\right).$$

As can be seen in the above equations, the polarization flip achieved by applying positive and negative electric field excitation of a bidirectional square wave can fully utilize the characteristics of high remnant polarization of the material, and the ECE obtained is over four times larger than that of the original electric field excitation method (for squared *P*-*E* hysteresis loops, $P_{r}\approx P_{m}$).

The advantages of polarization flip achieved by applying positive and negative electric field excitation of a bidirectional square wave are as follows:

1. The high remanent polarization of NFEs can be fully used for polarization flip to obtain an over four-fold enhancement in ECE ∆T and ∆S.
2. The full ferroelectric phase region can be employed for polarization flip, not just the FE-PE phase transition region. Then, the working temperature can be extended to the whole ferroelectric phase region.
3. High ∆T/∆E can be obtained due to the huge enhanced ECE ∆T. Here, in [110] PMN-PT single crystals, an ∆T/∆E over 2.50 K/(MV/m) was obtained from 290 K to 370 K, and the maximum Δ*T*/Δ*E* was 2.75 K/(MV/m).

Based on the “polarization flip” strategy, Lu et al. [90] carried out further explorations on a BT-based solution (see Figure 11g–l), e.g., 0.7BiFeO_3_-0.3BaTiO_3_-based material system, with a large $P_{r}$. The experimental results show that the sample with 0.05 wt% MnO_2_ added has a ∆T of 0.14 K under the conventional test method of applying a 3 MV/m external electric field for a period of time, and then removing the electric field. However, using the “polarization flip” strategy—that is, directly changing the external electric field from +3 MV/m to 0, and from 0 to −3 MV/m after applying the external electric field of −3 MV/m for a period of time, the sample shows a significant enhancement in the ECE, obtaining a ∆T of −0.56 K. Compared with the conventional ECE test method, the “polarization flip” strategy increases ∆T by nearly four times. This strategy also showed the great potential of NFE materials to be used for ECE applications.

## 6. Perspective

To realize the great potential of BT-based EC ceramics in practical solid-state refrigeration applications, several critical directions should be further explored.

1. Enhancing ECE via local structural engineering

Polarization entropy is a key factor governing the magnitude of the ECE. Thus, its enhancement represents a fundamental breakthrough toward high-performance EC materials. Recent investigations have shifted the focus from conventional domain structures to multiscale local configurations such as PNRs, domain engineering, and multi-phase coexistence. A critical challenge lies in how to precisely manipulate the cooperative evolution of these local structures under an applied electric field and/or a thermal stimulus to achieve simultaneous improvements in both polarization and polarization entropy. Traditional characterization techniques, such as scanning electron microscopy and laboratory X-ray diffraction, are insufficient to probe the dynamic evolution of local structures. Therefore, employing advanced in situ techniques—such as atomic pair distribution function analysis based on synchrotron or neutron total scattering, in situ transmission electron microscopy, and aberration-corrected electron microscopy will be essential. Moreover, combining these methods with theoretical approaches, such as first-principle calculations and multiscale phase-field simulation, will be instrumental in establishing a robust structure–property relationship linking the local structural evolution and the ECE.

2. Exploring the underlying mechanism of polarization flip

The polarization flip strategy, which involves the application of alternating positive and negative electric fields, has demonstrated the ability to enhance the ECE by over four-fold compared with conventional unidirectional excitation. More importantly, this approach breaks through the limitation of the phase transition regime, enabling effective ECE across a broader temperature range. However, the essential mechanism behind this remarkable enhancement remains unexplored. Future studies should focus on understanding the structure–property relationship underpinning polarization flip-induced ECE improvements, particularly through in situ electric field-dependent domain dynamics and energy dissipation analyses. Clarifying the correlation between the polarization switching behavior, energy–entropy transfer, and local defect/domain structure evolution will be the key to unlocking this strategy’s full potential.

3. Bridging material design and device integration.

A significant step toward applications lies in translating the excellent EC materials into viable device architectures. Among the promising designs, MLCCs with interdigitated electrodes have demonstrated great potential as EC cooling elements, owing to their high DBS and thermal response speed. For BT-based systems, the compatibility of their sintering temperature with nickel inner electrodes also offers a pathway for the fabrication of low-cost, lead-free MLCCs using base metal inner electrodes. It is noteworthy that cyclic stability is a key factor for practical EC devices. Weyland et al. [128] demonstrated that the ECE in Ba(Zr_0.2_Ti_0.8_)O_3_ decreased by only ~3% even after 10^6^ cycles, while Jiang et al. [129] reported a trivial degradation of <4.4% in Ba_0.85_Sr_0.15_Hf_0.06_Ti_0.94_O_3_ after 10^6^ cycles, confirming the excellent fatigue resistance of BT-based systems. The cyclic stability is generally associated with domain growth, defect pinning, and the suppression of pre-breakdown processes under repeated electric fields. Future research should emphasize device-level optimization, including thermal interface engineering, mechanical stability under cycling, and the development of scalable fabrication processes.

4. Employing machine learning (ML) tools to predict ECE.

With the compositions and structural characteristics of BT-based ceramics becoming increasingly well understood, machine learning (ML) offers a powerful approach to accelerate the prediction and design of more complex compositions in the future. By training models on existing datasets of chemical compositions, processing parameters, and measured ECE properties, ML can provide rapid screening of candidate materials, reveal hidden correlations between the microstructural features and the electrocaloric properties, and even guide the optimization of synthesis routes. Recent advances in combining ML with first-principle calculations and phase-field simulations further demonstrate the potential of data-driven methods to not only predict key characteristics such as phase structure, polarization dynamics, and electrocaloric strength, but also to propose unconventional design strategies beyond human intuition [91]. As the database of EC materials continues to expand, integrating ML into the discovery pipeline is expected to significantly shorten the period from material design to device application.

## Figures and Tables

**Table 1:** Summary of recently reported ECE in EC materials.

Composition	*E* (MV/m)	Temperature (℃)	∆T (K)	∆T/∆E (K·m/MV)	Methods	Ref.
0.5Ba(Sn_0.11_Ti_0.89_)O_3_-0.5Ba(Zr_0.15_Ti_0.85_)O_3_ three-layers	3	70.6	0.52	0.173	Indirect	[35]
(Ba_0.98125_La_0.0125_) (Sn_0.05_Nb_0.01_Ti_0.9375_)O_3_	5	RT	1.31	0.262	Indirect	[36]
(Ba_0.65_Sr_0.3_Ca_0.05_)(Sn_0.02_Ti_0.98_)O_3_	9	20	2.19	0.243	Direct	[37]
Ba_0.75_Sr_0.25_Zr_0.05_ (Ti_0.999_Mn_0.001_)_0.95_O_3_	3.5	70	1.3	0.371	Direct	[38]
<111>_c_-texture 0.955BaTiO_3_-0.045KNbO_3_	10	50	3.9	0.390	Direct	[39]
0.5Ba(Zr_0.2_Ti_0.8_)O_3_-0.5(Ba_0.7_Ca_0.3_)TiO_3_	8	50	2.5	0.313	Indirect	[40]
Ba_0.9925_La_0.005_Ti_0.9_Zr_0.1_O_3_	5	90	1.22	0.244	Direct	[41]
0.98Ba(Ti_0.9_Sn_0.1_)O_3_-0.02Bi(Mg_0.5_Ti_0.5_)O_3_	5	90	0.41	0.082	Direct	[42]
Ba_0.85_Sr_0.15_Ti_0.9_Hf_0.1_O_3_ -Ba_0.85_Sr_0.0725_Ca_0.0725_Ti_0.9_Hf_0.1_O_3_- Ba_0.85_Ca_0.15_Ti_0.9_Hf_0.1_O_3_ three-layers	5	80	1.2	0.240	Indirect	[43]
Ba_0.62_Ca_0.20_Sr_0.18_Sn_0.065_Ti_0.935_O_3_	5	40	0.49	0.098	Direct	[44]
(Ba_0.85_Ca_0.15_)(Ti_0.94_Hf_0.06_)O_3_	4	105	1.03	0.258	Indirect	[45]
0.3Ba_0.72_Sr_0.28_TiO_3_-0.5BaTi_0.8_Sn_0.2_O_3_-0.2Ba_0.72_Ca_0.28_TiO_3_	14	38	2.71	0.194	Indirect	[46]
Ba_0.8_Sr_0.2_Zr_0.15_(Ti_0.999_Mn_0.001_)_0.85_O_3_	5	70	1.08	0.216	Direct	[47]
(Ba_0.88_Ca_0.12_)(Ti_0.94_Sn_0.06_)O_3_	1.67	107	0.52	0.311	Indirect	[48]
0.6(Ba_0.7_Sr_0.3_)TiO_3_-0.4 Ba(Zr_0.2_Ti_0.8_)O_3_	3	69	0.53	0.177	Indirect	[49]
0.8BaTiO_3_–0.2Na_0.5_Bi_0.5_TiO_3_	4	184	1.65	0.413	Direct	[50]
Ba_0.95_Ca_0.05_Sn_0.09_Ti_0.91_O_3_	3	60	1.03	0.343	Indirect	[51]
Ba_0.8_Zr_0.2_TiO_3_	8	50	1.63	0.204	Direct	[52]
(Ba_0.9925_Sm_0.005_)(Ti_0⋅9_Sn_0.1_)O_3_	9	100	0.9	0.100	Indirect	[53]
Ba_0.9_Ca_0.1_Ti_0.85_Sn_0.15_O_3_	5	30	0.85	0.170	Direct	[54]
(Ba_0.985_La_0.01_)[Nb_0.028_(Ti_0.96_Sn_0.005_)]O_3_	5	53	1.14	0.228	Indirect	[55]
Ba_0.99_Dy_0.01_TiO_3_	5	120	1.92	0.384	Indirect	[56]
(Ba_0.97_Sm_0.02_)TiO_3_	3	72	1.11	0.370	Indirect	[57]
Ba_0.87_Ca_0.13_Ti_0.8955_Zr_0.0005_Zn_0.005/3_Nb_0.01/3_O_3_	3	65	0.701	0.234	Indirect	[58]
Ba(Ti_0.96_Sn_0.04_)O_3_	20	48.4	6.36	0.318	Indirect	[59]
(Ba_0.9_Sr_0.1_)(Hf_0.1_Ti_0.9_)O_3_	5	87	1.2	0.240	Indirect	[60]
Ba_0.85_Ca_0.15_Hf_0.10_Ti_0.90_O_3_	2	87	1.01	0.505	Indirect	[61]
BaZr_0.2_Ti_0.8_O_3_-5.7 mol % Li_2_CO_3_	15	60	2.26	0.151	Direct	[62]
1 mol% B^3+^ + 0.5 mol% Mn^2+^- Ba_0.7_Sr_0.3_TiO_3_	10	RT	3.08	0.308	Indirect	[63]
Ba_0.7_Ca_0.3_TiO_3_	3	127	0.419	0.140	Indirect	[64]
Ba(Hf_0.05_Sn_0.05_Zr_0.07_Ti_0.83_)O_3_	4	RT	1.7	0.425	Direct	[65]
Ba_0.82_Sr_0.18_Sn_0.065_Ti_0.935_O_3_	5	30	0.59	0.118	Indirect	[66]
(Ba_0.82_Ca_0.05_Sr_0.13_)(Ti_0.89_Zr_0.01_Sn_0.10_)O_3_	2	15	0.6	0.300	Direct	[67]
BaTi_0.89_Sn_0.11_O_3_-BaTi_0.85_Zr_0.15_O_3_-BaTi_0.89_Hf_0.11_O_3_ three-layers	2	84	0.64	0.320	Direct	[68]
<011>-oriented BaTiO_3_ single crystal	1.5	15	1.33	0.887	Direct	[69]
Ba_0.8_Sr_0.2_TiO_3_-1mol. % Mn^3+^	2	80	0.61	0.305	Indirect	[70]
Ba_0.9_Sr_0.1_Hf_0.1_Ti_0.9_O_3_-2 mol% CuO	3	RT	0.368	0.123	Direct	[71]
0.30BaHf_0.2_Ti_0.8_O_3_-0.7Ba_0.94_Sm_0.04_TiO_3_	3	64	0.46	0.153	Indirect	[72]
[0.94(Bi_0.5_Na_0.5_)TiO_3_–0.06BaTiO_3_]_200_/[0.5(Ba_0.7_Ca_0.3_)TiO_3_–0.5Ba(Zr_0.2_Ti_0.8_)O_3_]_200_ bilayers	62	97	23	0.371	Indirect	[73]
0.45BaZr_0.2_Ti_0.8_O_3_-0.55Ba_0.7_Ca_0.3_TiO_3_ single crystal	1.2	131	0.46	0.383	Indirect	[74]
BaTiO_3_ Single crystal	1.2	130	0.8	0.667	Direct	[75]
BaTi_0.998_Mn_0.002_O_3_	17	126	2.4	0.141	Direct	[76]
Ba(Ti_0.88_Sn_0.12_)O_3_	2	50	0.27	0.135	Indirect	[77]
Ba(Zr_0.2_Ti_0.8_)O_3_	14.5	39	4.5	0.310	Direct	[79]
Ba(Hf_0.11_Ti_0.89_)O_3_	5	65	1.35	0.270	Indirect	[80]
(Ba_0.78_Ca_0.22_)(Ti_0.88_Sn_0.12_)O_3_	2.5	40	0.5	0.200	Indirect	[81]
Ba(Sn_0.014_Ti_0.186_)O_3_-Ba(Hf_0.08_Ti_0.72_)O_3_	7	80	3.35	0.479	Direct	[18]
Ba(Zr_0.15_Mn_0.0025_Ti_0.8475_)O_3_	5	62	2.35	0.470	Direct	[82]
Ba_0.6_Sr_0.4_Mn_0.001_Ti_0.999_O_3_	5	21	2.75	0.550	Direct	[83]
Ba_0.7_Sr_0.3_TiO_3_	4	40	2.18	0.545	Direct	[84]
Na_0.2_Bi_0.2_Ba_0.2_Sr_0.2_Ca_0.2_TiO_3_	6	40	0.63	0.105	Indirect	[85]
(Bi_1/6_Na_1/6_Sr_1/6_Ba_1/6_Pb_1/6_Ca_1/6_)TiO_3_	6	140	0.63	0.105	Direct	[86]
(Bi_1/6_La_1/6_Na_1/6_K_1/6_Sr_1/6_Ba_1/6_)TiO_3_	6	RT	0.14	0.023	Indirect	[86]
Ba_0.85_Ca_0.15_Ti_0.85_Zr_0.15_O_3_	14	40	1.78	0.127	Indirect	[87]
Ba_0.65_Sr_0.35_TiO_3-_0.5 wt% B_2_O_3_-ZnO	5	10	2.29	0.458	Direct	[88]
Ba(Zr_0.2_Ti_0.8_)O_3_	19.5	40	7	0.359	Indirect	[89]
0.7BiFeO_3_-0.3BaTiO_3-_0.05 wt% MnO_2_	3	21.6	0.56	0.187	Direct	[90]
BaTi_0.88_Hf_0.12_O_3_	4.8	67	1.284	0.268	Indirect	[91]

**Table 2:** The radii of A-site ions.

Ion	CN	Radius (Å)
Ba^2+^	12	1.61
Li^+^		1.18
Na^+^		1.39
K^+^		1.64
Ca^2+^		1.34
Sr^2+^		1.44
Pb^2+^		1.49
Bi^3+^		1.32
La^3+^		1.36
Ce^3+^		1.34
Sm^3+^		1.24

**Table 3:** The radii of B-site ions.

Ion	CN	Radius (Å)
Ti^4+^	6	0.605
Zr^4+^		0.72
Sn^4+^		0.69
Hf^4+^		0.71
Ce^4+^		0.87
Mg^2+^		0.72
Mn^2+^		0.83
Mn^3+^		0.645
Mn^4+^		0.53
Y^3+^		0.9
Ta^5+^		0.64
Nb^5+^		0.64

## Data Availability

No new data were created or analyzed in this study. Data sharing is not applicable to this article.
